# Cost‐effectiveness of one month of daily isoniazid and rifapentine versus three months of weekly isoniazid and rifapentine for prevention of tuberculosis among people receiving antiretroviral therapy in Uganda

**DOI:** 10.1002/jia2.25623

**Published:** 2020-10-18

**Authors:** Olivia Ferguson, Youngji Jo, Jeff Pennington, Karl Johnson, Richard E Chaisson, Gavin Churchyard, David Dowdy

**Affiliations:** ^1^ Department of Epidemiology Johns Hopkins Bloomberg School of Public Health Baltimore MD USA; ^2^ Department of Medicine Center for Tuberculosis Research Johns Hopkins University School of Medicine Baltimore MD USA; ^3^ Aurum Institute Parktown South Africa; ^4^ School of Public Health University of Witwatersrand Johannesburg South Africa

**Keywords:** tuberculosis, preventive therapy, isoniazid, rifapentine, cost‐effectiveness analysis, short‐course treatment

## Abstract

**Introduction:**

Preventive therapy is essential for reducing tuberculosis (TB) burden among people living with HIV (PLWH) in high‐burden settings. Short‐course preventive therapy regimens, such as three‐month weekly rifapentine and isoniazid (3HP) and one‐month daily rifapentine and isoniazid (1HP), may help facilitate uptake of preventive therapy for latently infected patients, but the comparative cost‐effectiveness of these regimens under different conditions is uncertain.

**Methods:**

We used a Markov state‐transition model to estimate the incremental costs and effectiveness of 1HP versus 3HP in a simulated cohort of patients attending an HIV clinic in Uganda, as an example of a low‐income, high‐burden setting in which TB preventive therapy might be prescribed to PLWH. Our primary outcome was the incremental cost‐effectiveness ratio, expressed as 2019 US dollars per disability‐adjusted life year (DALY) averted. We estimated cost‐effectiveness under different conditions of treatment completion and efficacy of 1HP versus 3HP, latent TB prevalence and rifapentine price.

**Results:**

Assuming equivalent clinical outcomes using 1HP and 3HP and a rifapentine price of $0.21 per 150 mg, 1HP would cost an additional $4.66 per patient treated. Assuming equivalent efficacy but 20% higher completion with 1HP versus 3HP, 1HP would cost $1,221 per DALY averted relative to 3HP. This could be reduced to $18 per DALY averted if 1HP had 5% greater efficacy than 3HP and the price of rifapentine were 50% lower. At a rifapentine price of $0.06 per 150 mg, 1HP would become cost‐neutral relative to 3HP.

**Conclusions:**

1HP has the potential to be cost‐effective under many realistic circumstances. Cost‐effectiveness depends on rifapentine price, relative completion and efficacy, prevalence of latent TB and local willingness‐to‐pay.

## 1 Introduction

Tuberculosis (TB) is the leading cause of death among people living with HIV (PLWH), and the leading single‐agent infectious cause of death worldwide [1, 2]. TB preventive therapy for latently infected patients has consistently been shown to reduce the incidence of active TB [3] and reduced the hazard of all‐cause mortality among PLWH by 37% in the Temprano ANRS 12136 trial [4]. As such, TB preventive therapy is universally recommended for PLWH as secondary prevention to prevent the reactivation of active TB among those with existing latent TB infection [5]. Despite these clear recommendations, TB preventive therapy remains underutilized, especially in high‐burden settings – partially due to the cost and logistical challenges of delivering TB preventive therapy at scale [6].

Six to nine months of daily isoniazid preventive therapy (IPT) has been the standard of care for TB preventive therapy for decades, but this regimen has been difficult to scale up due in part to low completion levels, as well as its non‐negligible toxicity [7, 8]. In 2011, a shorter‐course regimen consisting of three months of weekly rifapentine and isoniazid (3HP) was demonstrated to be as effective as IPT with lower rates of liver toxicity and higher rates of treatment completion; 3HP has since been recommended as an alternative to IPT, but still requires three months of treatment and a large pill burden on dosing days [9, 10]. Recently, a one‐month regimen of daily isoniazid and rifapentine (1HP) was shown to be non‐inferior to IPT among people living with HIV [11]. However, the conditions required for this regimen – which requires a larger total dose of rifapentine (due to daily rather than weekly dosing) – to be cost‐effective remain uncertain. The aim of this analysis was to determine the conditions under which 1HP may be considered cost‐effective relative to 3HP, when delivered to patients in a typical HIV clinic in Uganda (as a representative high‐burden setting).

## 2 Methods

We use a typical HIV clinic in Uganda as a reference point for this analysis, which we perform from a health system perspective. Our primary outcome was the incremental cost‐effectiveness ratio (ICER), expressed in 2019 US dollars per disability‐adjusted life year (DALY) averted. This modelling exercise is based on previously published literature and does not involve any human subjects research.

### 2.1 Model structure

**Figure 1:**
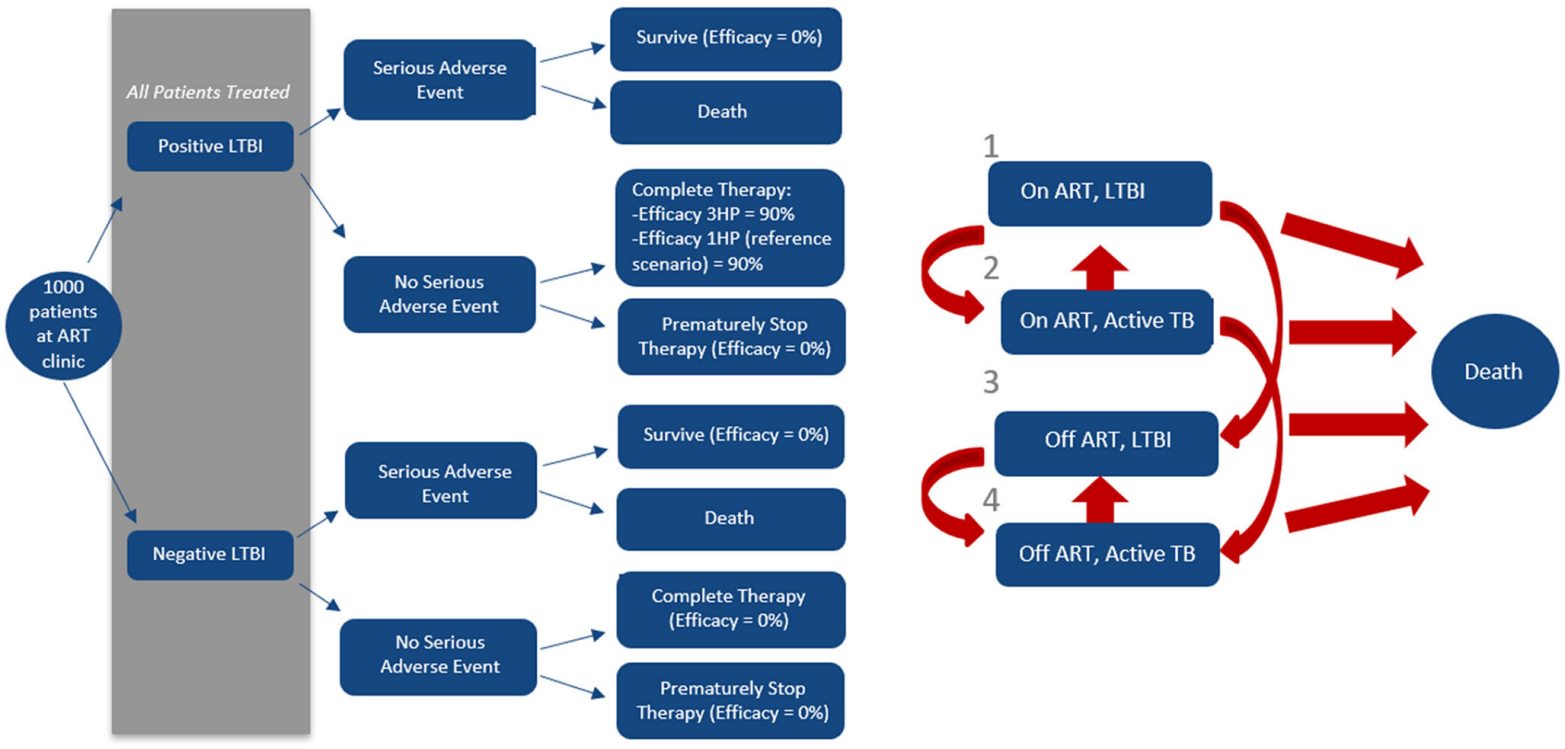
Model structure. We first model TB preventive therapy treatment outcomes among 1000 people being treated for HIV, as shown in the decision tree on the left. During this process, we consider probabilities of experiencing treatment‐limiting adverse events (a small fraction of which may result in death) and of treatment success among individuals who do not experience adverse events. We model four outcomes, based on LTBI status (positive or negative) and TB preventive therapy completion status (yes/no). Patients in each group are then followed for 20 years, during which time they experience transitions that include TB reactivation (with subsequent treatment and mortality risk) and disengagement and re‐engagement in HIV care (antiretroviral therapy, ART). Outcomes are then summed over 1000 patients and compared between different scenarios of TB preventive therapy.

We adapted a previously published model of cost‐effectiveness of preventive therapy for tuberculosis in Uganda (Figure 1) [12]. In brief, this model simulates 1000 patients being treated for HIV and initiating TB preventive therapy (1HP or 3HP) over a horizon of 20‐years under different assumptions about the delivery of TB preventive therapy. Patients are characterized as having or not having underlying latent tuberculosis infection (LTBI), but we assume that, in keeping with international guidelines, preventive therapy is given irrespective of LTBI status (i.e. no test for LTBI performed prior to TB preventive therapy initiation). After starting TB preventive therapy, a proportion of patients is assumed to experience adverse events of sufficient severity to warrant cessation of therapy, and a proportion who do not experience such adverse events are assumed to complete treatment. In the absence of data on the relationship between doses taken and preventive efficacy, we conceptualize “completion” as a weighted average of therapeutic doses taken. Thus, for example, two patients who each take half of the prescribed regimen are assumed to have the same outcomes as one patient who takes a full course plus one who takes no TB preventive therapy (50% completion in both scenarios). Upon finishing the preventive therapy phase, simulated individuals are followed for 20 years to track future events of TB reactivation. We assume that the effect of TB preventive therapy is to reduce the risk of reactivation TB among those who complete treatment, and that this effect is permanent – this can be conceptualized as curing a proportion of individuals with LTBI commensurate with (completion × efficacy), while still allowing reinfection (which is unaffected by TB preventive therapy) to occur throughout the 20‐year follow‐up period. Individuals without LTBI are assumed to have no risk of reactivation (and thus receive no benefit from TB preventive therapy). We further assume that all individuals have an equal risk of future TB reinfection, and that this risk is not affected by LTBI status at baseline or the completion of preventive therapy. We also account for disengagement from ART in the first three years of the model, under the assumption that patients who are likely to disengage from ART care will do so in the first three years (or alternatively that, after three years, net disengagement is equal to net re‐engagement). DALYs are calculated by assigning disability weights to the time spent in each Markov state (with disutility experienced due to both HIV and active TB) to calculate years of life with disability (YLD) and adding this to years of life lost (YLL), calculated as the discounted life expectancy at the time of death for individuals who die prior to the 20‐year time horizon regardless of cause. YLL beyond the 20‐year horizon are not included, as a conservative assumption given the uncertainty of events (e.g. innovations in TB or HIV management) that may occur beyond that time horizon. Primary model parameter values are provided in Table 1.

**Table 1:** Model parameters

Parameter description	Model value	Low value	High value
Epidemiologic and health system values			
Prevalence of LTBI [13]	0.261	0.111	0.397
Rate of disengagement from HIV care, per year [14]	0.108	0.073	0.146
Proportion completing 3HP [9]	0.74	0.47	0.89
Efficacy of 3HP [9]	0.90	0.77	1
Mortality (annual risk)			
HIV‐positive, on ART [15]	0.0354	0.0254	0.047
HIV‐positive, off ART [16]	0.1326	0.1242	0.1462
Active TB, on ART, receiving treatment for TB [17, 18]	0.1	0.05	0.179
Active TB, Off ART, not receiving treatment for TB [19]	0.81	0.07	0.99
Morbidity			
Annual Risk of TB Reactivation for PLWH, no ART or TB Preventive Therapy [20]	0.043	0.037	0.049
Relative risk of TB reactivation while on ART [21]	0.35	0.28	0.44
Prevalence of a nonlethal adverse event during TB preventive therapy [13, 22, 23]	0.034	0.018	0.049
Disability weights			
Off ART, LTBI [24]	0.582	0.406	0.743
On ART, LTBI [24]	0.078	0.052	0.111
Active TB [24]	0.408	0.274	0.549
Costs (2019 US dollars)			
Price of Rifapentine (per 150m g) [25]	$0.21	$0.19	$0.27
Price of Isoniazid (per 150 mg)	$0.02	N/A	N/A
Cost of outpatient Visit [26]	$1.41	$1.08	$8.15
Yearly cost of ART drugs [27]	$191.81	$169	$212
Yearly cost of Active TB treatment [28]	$231.02	$180	$280

1HP, 1 month (28 doses) of daily isoniazid and rifapentine; 3HP, 3 months (12 doses) of weekly isoniazid and rifapentine; ART, antiretroviral therapy; DALYs, disability‐adjusted life years; HIV, human immunodeficiency virus; TB, tuberculosis.

### 2.2 Costs

Model costs are evaluated from a health system perspective. These include the price of drugs for TB preventive therapy (1HP or 3HP), the unit cost of outpatient visits, the annual cost of ART, and the cost of active TB treatment (averted cost due to effective preventive therapy). The price of rifapentine was assumed to be $0.21 per 150 mg, as announced by Unitaid, Sanofi and the Global Fund on 31 October 2019 [25]. We incorporated a 10% markup in this price to account for the costs of procurement and delivery. Patients who do not successfully complete the full course of preventive therapy do not incur the full cost of the regimen (see Table S1). Model costs are reported in US Dollars and inflated to the year 2019 using the US Consumer Price Index [29]. Costs and effects were both discounted at a rate of 3% per year.

### 2.3 Analysis

Given the uncertainty around the values of key parameters that play a major role in determining the incremental cost‐effectiveness of 1HP versus 3HP, we estimated incremental cost‐effectiveness across a range of scenarios under different values of four key factors: incremental efficacy of 1HP, incremental completion of 1HP, price of rifapentine, and prevalence of LTBI in the population receiving TB preventive therapy.

- Difference in 1HP Efficacy: Efficacy is defined as the probability of successfully eliminating the risk of TB reactivation after completing a full course of TB preventive therapy. We consider the efficacy of 3HP to be 0.90 [9]. The difference in efficacy of 1HP was therefore taken as (efficacy of 1HP – 0.90). In the reference scenario, we assumed equivalent efficacy for 1HP and 3HP (i.e. difference in efficacy of 0), but we explored scenarios in which completing one month (28 doses) of 1HP would have up to 0.10 higher absolute efficacy than completing three months (12 doses) of 3HP. However, it is worth noting that the original study suggests 1HP likely carries greater efficacy than 3HP, further plausible due to the regimen’s higher exposure to rifapentine.
- Difference in 1HP Completion: Completion of preventive therapy is defined as a weighted average of the total number of doses completed, as above. For simplicity of analysis, we assume that the proportional reduction in TB reactivation risk achieved through TB preventive therapy delivery is equal to (efficacy × completion). In the reference scenario, we assumed 0.74 completion of 3HP and 0.94 completion of 1HP, such that the difference in completion of 1HP was 0.20. Given the absence of comparative data to inform this parameter value in field settings, we explored differences in completion values between 0 (0.74 completion of 1HP) and 0.25 (0.99 completion of 1HP).
- Price of Rifapentine: We took as our reference value the recently announced price of rifapentine of $0.21 per 150 mg, corresponding to $15 per patient course of 3HP and $24 per course of 1HP, and inflated this by 10% for procurement and delivery as above. We considered a further reduction in price to $0.12 per 150 mg and also evaluated the price point at which 1HP and 3HP would become cost‐neutral.
- LTBI Prevalence: We took as our reference value the estimated LTBI prevalence in Uganda of 0.26 [13]. We considered scenarios of low (0.20), medium (0.50) and high (0,80) LTBI prevalence. This represents the prevalence of LTBI within the population of PLWH receiving TB preventive therapy, not the general population; thus, high LTBI prevalence could correspond to a population that tested positive on tuberculin skin testing or interferon‐gamma release assay (IGRA).

We conducted a series of multivariate analyses to quantify relationships between the values of each of these parameters and the estimated incremental cost‐effectiveness of 1HP, under different cost‐effectiveness thresholds. We also conducted a probabilistic sensitivity analysis to explore uncertainty within the model parameter values, and define the range within which 95% of model outcomes fall.

## 3 Results

We estimated that, in our representative setting of a Ugandan HIV clinic, 1000 PLWH taking 3HP would complete 715 courses of treatment. Over the ensuing 20 years, assuming an LTBI prevalence of 0.26 and an annual risk of TB reactivation of 1.5% among those not completing TB preventive therapy but remaining on ART, an estimated 21 cases of TB reactivation and 7 TB deaths would occur – primarily among the 74 individuals with LTBI who did not complete TB preventive therapy (Table 2). Assuming 1HP and 3HP have equal efficacy and are completed to equivalent degrees (i.e. no difference in outcomes), 1HP would cost an Incremental $4.66 per patient relative to 3HP ($4,655 per 1000 patients, Table 3). Under the (reference) scenario in which 1HP has similar efficacy but results in 0.20 greater absolute completion relative to 3HP (e.g. 0.94 vs. 0.74), the incremental cost‐effectiveness of 1HP was estimated at $1,221 per DALY averted. Assuming a 0.05 difference in efficacy of 1HP (e.g. 0.95 vs. 0.90, in addition to the 0.20 difference in completion), the estimated cost‐effectiveness of 1HP improved to $893 per DALY averted; if combined with a 50% reduction in the price of rifapentine, the incremental cost‐effectiveness of 1HP relative to 3HP could be as low as $18 per DALY averted. Under our baseline assumptions, 1HP would become cost‐neutral relative to 3HP if the price of rifapentine fell to $0.06 per 150 mg, although this is unlikely to occur as this price is less than the current price of six months of isoniazid (6H).

**Table 2:** Outcomes, per 1000 individuals being treated for HIV initiating TB preventive therapy

	Total cost	Preventive therapy courses completed	Cases of TB Reactivation	TB deaths	DALYs	Incremental cost‐effectiveness ratio (USD per DALY averted)^a^
1. Equivalent efficacy/completion						
1HP	$1 526 664	714.8	21.3	6.7	7396.7	
3HP	$1 522 009	714.8	21.3	6.7	7396.7	
Incremental	$4655	0.0	0.0	0.0	0.0	N/A
2. 0.20 Difference in 1HP completion (reference scenario)						
1HP	$1 528 168	908.0	11.1	3.6	7391.7	
3HP	$1 522 009	714.8	21.3	6.7	7396.7	
Incremental	$6159	193.2	10.2	3.1	5.0	$1221 ($845, $1424)
3. 0.20 Difference in 1HP completion Rate, 0.05 difference in IHP efficacy						
1HP	$1 527 723	908.0	8.3	2.6	7390.3	
3HP	$1 522 009	714.8	21.3	6.7	7396.7	
Incremental	$5714	193.2	13.1	4.1	6.4	$893 ($822, $1287)
4. 0.20 Difference in 1HP completion rate, 0.05 difference in 1HP efficacy, 50% reduced rifapentine price						
1HP	$1 515 389	908.04	8.3	2.6	7390.3	
3HP	$1 515 277	714.84	21.3	6.7	7396.7	
Incremental	$112	193.2	13.1	4.1	6.4	$18 ($10, $517)

1HP, 1 month (28 doses) of daily isoniazid and rifapentine; 3HP, 3 months (12 doses) of weekly isoniazid and rifapentine; TB, tuberculosis; DALYs, disability‐adjusted life years.

^a^ Incremental cost‐effectiveness expressed in 2019 US dollars per disability‐adjusted life‐year averted.

**Table 3:** Cost components

	1HP	3HP	Difference (1HP − 3HP)
1. Equivalent efficacy/completion			
Cost of preventive treatment	$27 377	$22 722	$4 655
Cost of active TB treatment	$3 894	$3 894	$0
Cost of ART/HIV care	$1 495 677	$1 495 393	$0
Total costs	$1 528 168	$1 522 009	$4 655
2. 0.20 Difference in 1HP completion (reference scenario)			
Cost of preventive treatment	$30 464	$22 722	$7 742
Cost of active TB treatment	$2 028	$3 894	‐$1 866
Cost of ART/HIV care	$1 495 677	$1 495 393	$284
Total costs	$1 528 168	$1 522 009	$6 159
3. 0.20 Difference in 1HP completion Rate, 0.05 difference in IHP efficacy			
Cost of preventive treatment	$30 464	$22 722	$7 742
Cost of active TB treatment	$1 507	$3 894	‐$2 388
Cost of ART/HIV CARE	$1 495 752	$1 495 393	$360
Total costs	$1 527 723	$1 522 009	$5 714
4. 0.20 Difference in 1HP completion rate, 0.05 difference in 1HP efficacy, 50% reduced rifapentine price			
Cost of preventive treatment	$18 130	$15 990	$2 140
Cost of active TB treatment	$1 506	$3 894	‐$2 388
Cost of ART/HIV care	$1 495 752	$1 495 393	$360
Total costs	$1 515 389	$1 515 277	$112

1HP, 1 month (28 doses) of daily isoniazid and rifapentine; 3HP, 3 months (12 doses) of weekly isoniazid and rifapentine; ART, antiretroviral therapy; DALYs, disability‐adjusted life years; HIV, human immunodeficiency virus; TB, tuberculosis.

**Figure 2:**
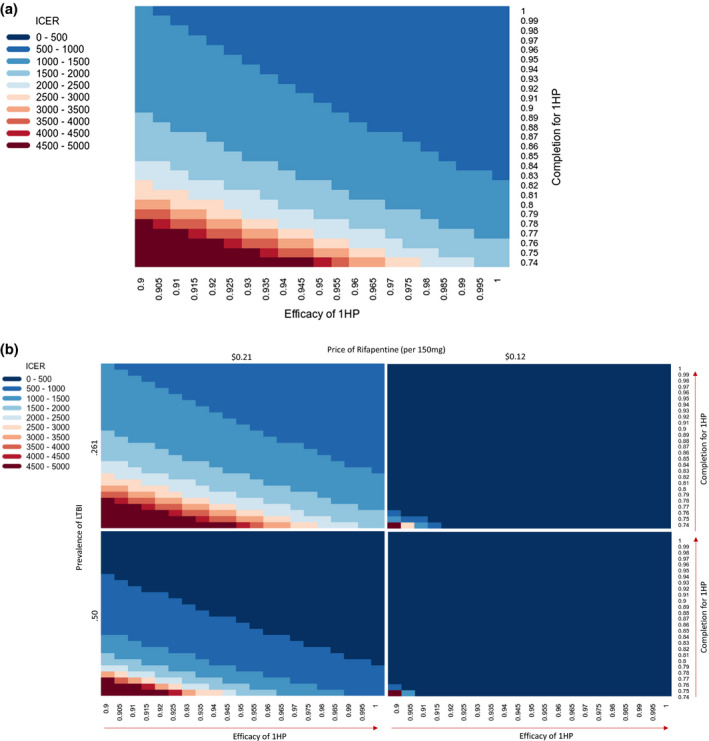
Multivariate analyses: incremental cost‐effectiveness of 1HP versus 3HP under different assumptions of 1HP completion and efficacy, price of Rifapentine, and LTBI prevalence. **(A)** In this two‐way sensitivity analysis, we varied the probability of completing 1HP on the y‐axis and the efficacy of 1HP on the x‐axis, holding all other model variables constant. Different colours represent different incremental cost‐effectiveness ratios, comparing 1HP to 3HP, under a scenario of 0.26 LTBI prevalence and price of rifapentine of $0.21 per 150 mg. For example, assuming equivalent efficacy of 1HP and 3HP but an absolute 20% increase in completion (from 0.74 to 0.94) with 1HP, the incremental cost‐effectiveness of 1HP was estimated at $1221 per disability‐adjusted life year (DALY) averted. Assuming an additional 5% increase in efficacy (from 0.9 to 0.95) caused this estimate to fall to $893 per DALY averted. At a cost‐effectiveness threshold of $1500 per DALY averted, all regions shaded in the darkest two shades of blue represent conditions under which 1HP would be considered cost‐effective relative to 3HP under the primary assumptions of the model. **(B)** The following figure shows a series of four two‐way sensitivity analyses similar to that shown in Part A, but under different assumptions regarding the price of rifapentine ($0.21 per 150 mg in the left panels, $0.12 per 150 mg in the right panels) and the prevalence of LTBI in the population (0.26 in the upper panels, 0.50 in the lower panels). The top left panel represents the reference scenario, also shown in Part A. As in Part A, blue represents scenarios where 1HP is more cost‐effective relative to 3HP, and red represents scenarios of diminishing cost‐effectiveness of 1HP.

Figure 2 expands these results by illustrating the estimated incremental cost‐effectiveness of 1HP relative to 3HP under a wide array of assumed values for the difference in efficacy of 1HP, difference in completion under 1HP, price of rifapentine, and prevalence of LTBI. For example to be cost‐effective at a threshold of $1,500 per DALY averted, 1HP would need to achieve an incremental absolute completion of 0.16 in a setting with 0.26 LTBI prevalence, assuming equivalent efficacy and the current price of rifapentine. The required difference in completion of 1HP to achieve cost‐effectiveness could fall to 0.07 in a similar setting with 0.50 LTBI prevalence, to 0.08 if the difference in efficacy was 0.05, to 0.01 if the price of rifapentine was reduced by half, and to 0.15 if the cost‐effectiveness threshold was increased to $2000 per DALY averted. Contours in Figure 2 illustrate the conditions that would need to be achieved for 1HP to be cost‐effective under a variety of cost‐effectiveness thresholds from $500/DALY averted to $5000/DALY averted. Probabilistic sensitivity analysis confirmed that uncertainty in other parameter values had relatively less effect on incremental cost‐effectiveness estimates than did variation in 1HP effectiveness and completion, as well as the price of rifapentine (Table 2, right column, vs. Figure 2; see also Appendix Section III).

## 4 Discussion

This analysis explores the conditions under which 1HP versus 3HP might be considered cost‐effective for the prevention of TB reactivation among people living with HIV in a typical Ugandan HIV clinic, from a health system perspective. These results extend earlier findings regarding the cost‐effectiveness of 3HP to a new promising regimen for TB prevention among PLWH. This analysis demonstrates that 1HP is likely to be cost‐effective relative to 3HP under a variety of scenarios – for example assuming equivalent efficacy of the two regimens, an LTBI prevalence of 0.26, and no change in the price of rifapentine, the rate of completion for 1HP would need to be 0.26 higher than that of 3HP to be cost‐effective at a threshold of $1000 per DALY averted (0.11 higher at a threshold of $2000 per DALY averted). If the price of rifapentine can be reduced in the future, the cost‐effectiveness of 1HP is likely to improve even further. These results may be useful to decision‐makers in understanding the relative cost‐effectiveness of different short‐course regimens for the prevention of TB among people living with HIV.

In evaluating whether health interventions are cost‐effective, there is no consensus as to the appropriate cost‐effectiveness threshold that should be used in a given context. Classical guidelines recommended the use of one to three times per‐capita gross domestic product per DALY averted as “cost‐effective” or “highly cost‐effective”, but this approach has recently been challenged as alternatively underestimating the value of a statistical life or overestimating ability/willingness to pay at the country level [30, 31, 32]. More recent cost‐effectiveness analyses in resource‐limited settings have tended to adopt more conservative thresholds based on the cost‐effectiveness of funded interventions (e.g. $500 per DALY averted for antiretroviral therapy in sub‐Saharan Africa or $161 per DALY averted for first‐line treatment for TB in Tanzania [33, 34]). Ultimately, cost‐effectiveness thresholds are inherently subjective, setting‐specific, and likely to change over time; as such, the present analysis cannot be interpreted as suggesting that 1HP is universally “more cost‐effective” than 3HP – as any such statement must be made in reference to a corresponding cost‐effectiveness threshold. Nevertheless, understanding the conditions under which 1HP would be considered cost‐effective, across a range of cost‐effectiveness thresholds, can be informative to decision making in a variety of high‐burden contexts.

Should the price of rifapentine be reduced to $0.06 per 150 mg, this analysis suggests that 1HP would be cost‐neutral relative to 3HP. In this case, as long as 1HP is expected to be equally effective (efficacy × completion), it would be the preferred regimen for this population. Even above this cost‐neutral threshold, the price of rifapentine remains the most important intervenable determinant of 1HP cost‐effectiveness. This analysis therefore supports efforts to lower the price of rifapentine, for purposes of making the 1HP regimen both more affordable and more cost‐effective. The cost‐effectiveness of 1HP is also optimized in populations with high LTBI prevalence – for example in settings where testing for LTBI is routine and TB preventive therapy only offered to people testing positive. The importance of incremental efficacy and incremental completion to estimating the cost‐effectiveness of 1HP argues for additional data (e.g. long‐term follow‐up of PLWH receiving 1HP and implementation trials comparing completion of 1HP vs. 3HP under routine conditions) to inform the value of these parameters under real‐world conditions.

As with any modelling study, this analysis is not without limitations. First, in the absence of existing empirical data to inform a true reference case in a specific setting, we instead evaluated cost‐effectiveness under a range of assumptions regarding the relative efficacy and completion of 1HP versus 3HP, and the prevalence of LTBI. These data can be useful for initial decision making but cannot replace the importance of data‐informed cost‐effectiveness analyses in specific settings once empirical data to inform these parameters become available. Second, to increase interpretability, we intentionally used a simplified model that does not incorporate detailed complexities of HIV or TB natural history; we also assumed no difference in adverse events. These assumptions make our findings more transparent and easy to understand from the perspective of decision‐makers but may also be less realistic when applied to any specific epidemiological and economic setting. Third, we modelled a population of PLWH engaged in care in a Ugandan HIV clinic, as a representative high‐burden setting in sub‐Saharan Africa; these results may not generalize to other populations (including HIV‐negative populations) or settings that differ meaningfully in their TB natural history or their epidemiological/economic conditions.

## 5 Conclusions

In summary, under the newly announced price of rifapentine, we estimated that 1HP would cost an additional $4.66 per patient relative to 3HP under assumptions of equal efficacy and completion. If completion were 20% higher (on an absolute scale) for 1HP, the cost‐effectiveness of this regimen relative to 3HP was estimated at $1,221 per DALY averted as delivered to a population of PLWH engaged in care in a Ugandan HIV clinic. The cost‐effectiveness of 1HP relative to 3HP is strongly driven by the price of rifapentine, LTBI prevalence, the difference in completion rates, and the difference in efficacy. We illustrate the conditions under which 1HP would be considered cost‐effective relative to 3HP at different cost‐effectiveness thresholds; this analysis also strongly supports further reductions in the price of rifapentine as a way to further enhance the cost‐effectiveness of 1HP. Short‐course TB preventive therapy is highly efficacious, carries low toxicity, and has the potential to avert tremendous morbidity and mortality; this analysis suggests that such regimens are likely to be highly cost‐effective for preventing active TB among PLWH in high TB‐burden countries when delivered under reasonable combinations of conditions with regard to drug efficacy (90% to 95%), regimen completion (85% to 95%), population LTBI prevalence (26% to 50%) and rifapentine price ($0.12‐$0.21 per 150 mg).

## Competing Interest

The authors of this paper report there are no conflicts of interest to report at this time.

## Authors’ Contributions

O.F., K.J., D.D, Y.J. and J.P. contributed to the design and building of the model. O.F. wrote the paper. D.D., Y.J., J.P., R.C. and G.C., reviewed and provided feedback on the paper.

## Supporting information


**Appendix S1.** The purpose of this appendix is to provide supplementary information on the model structure and the analysis performed.

**Table S1.** Complete versus incomplete preventive regimen costs
**Table S2.** Parameter distributions for probabilistic sensitivity analysis
**Figure S1.** One‐way sensitivity analysis, impact on ICER (2019 USD per DALYs averted).
**Figure S2.** Probabilistic sensitivity analyses.

## References

[1] Tuberculosis (TB) [cited 2019 Dec 11]. Available from: https://www.unaids.org/en/topic/tuberculosis

[2] MacNeil A , Glaziou P , Sismanidis C , Maloney S , Floyd K . Global epidemiology of tuberculosis and progress toward achieving global targets — 2017. Morb Mort Wkly Rep. 2019;68(11):263–6. 10.15585/mmwr.mm6811a3 PMC6478060 30897077

[3] Ayele HT , van Mourik MSM , Debray TPA , Bonten MJM . Isoniazid prophylactic therapy for the prevention of tuberculosis in HIV infected adults: a systematic review and meta‐analysis of randomized trials. PLoS One. 2015;10(11):e0142290 26551023 10.1371/journal.pone.0142290 PMC4638336

[4] Badje A , Moh R , Gabillard D , Guéhi C , Kabran M , Ntakpé JB , et al. Effect of isoniazid preventive therapy on risk of death in west African, HIV‐infected adults with high CD4 cell counts: long‐term follow‐up of the Temprano ANRS 12136 trial. Lancet Global Health. 2017;5(11):e1080–9. 29025631 10.1016/S2214-109X(17)30372-8

[5] World Health Organization . Guidelines on the management of latent tuberculosis infection. Geneva, Switzerland: WHO; 2015. 25973515

[6] Global tuberculosis report 2019. Geneva: World Health Organization; 2019. Licence: CC BY‐NC‐SA 3.0 IGO.

[7] Smieja MJ , Marchetti CA , Cook DJ , Smaill FM . Isoniazid for preventing tuberculosis in non‐HIV infected persons. Cochrane Database Syst Rev. 2000;CD001363. 10.1002/14651858.CD001363 PMC6532737 10796642

[8] LoBue PA , Moser KS . Use of isoniazid for latent tuberculosis infection in a public health clinic. Am J Respir Crit Care Med. 2003;168:443–7. 12746255 10.1164/rccm.200303-390OC

[9] Sterling TR , Villarino ME , Borisov AS , Shang N , Gordin F , Bliven‐Sizemore E , et al. Three months of rifapentine and isoniazid for latent tuberculosis infection. N Engl J Med. 2011;365:2155–66. 22150035 10.1056/NEJMoa1104875

[10] Latent tuberculosis infection: updated and consolidated guidelines for programmatic management. Geneva: World Health Organization, 2018. Contract No.: WHO/CDS/TB/2018.4. 30277688

[11] Swindells S , Ramchandani R , Gupta A , Benson CA , Leon‐Cruz J , Mwelase N , et al. One month of Rifapentine plus Isoniazid to prevent HIV‐related tuberculosis. N Engl J Med. 2019;380( 11):1001–11. 30865794 10.1056/NEJMoa1806808 PMC6563914

[12] Johnson KT , Churchyard GJ , Sohn H , Dowdy DW . Cost‐effectiveness of preventive therapy for tuberculosis with isoniazid and rifapentine versus isoniazid alone in high‐burden settings. Clin Infect Dis. 2018;67(7):1072–1078. 29617965 10.1093/cid/ciy230

[13] Houben RM , Dodd PJ . The global burden of latent tuberculosis infection: a re‐estimation using mathematical modelling. PLoS Medicine. 2016;13:e1002152. 27780211 10.1371/journal.pmed.1002152 PMC5079585

[14] Fox MP , Rosen S . Patient retention in antiretroviral therapy programs up to three years on treatment in sub‐Saharan Africa, 2007‐2009: systematic review. Trop Med Int Health. 2010;2009:1–15. 10.1111/j.1365-3156.2010.02508.x PMC2948795 20586956

[15] Mills EJ , Bakanda C , Birungi J , Chan K , Ford N , Cooper CL , et al. Life expectancy of persons receiving combination antiretroviral therapy in low‐income countries: a cohort analysis from Uganda. Ann Intern Med. 2011;155:209–16. 21768555 10.7326/0003-4819-155-4-201108160-00358

[16] Sewankambo NK , Gray RH , Ahmad S , Serwadda D , Wabwire‐Mangen F , Nalugoda F , et al. Mortality associated with HIV infection in rural Rakai District, Uganda. AIDS. 2000;14:2391–400. 11089628 10.1097/00002030-200010200-00021

[17] Moore D , Liechty C , Ekwaru P , Were W , Mwima G , Solberg P , et al. Prevalence, incidence and mortality associated with tuberculosis in HIV‐infected patients initiating antiretroviral therapy in rural Uganda. AIDS. 2007;21:713–9. 17413692 10.1097/QAD.0b013e328013f632

[18] World Health Organization . 2016 Global tuberculosis report. Geneva, Switzerland: WHO; 2016.

[19] Corbett EL , Watt CJ , Walker N , Maher D , Williams BG , Raviglione MC , et al. The growing burden of tuberculosis: globaltrends and interactions with the HIV epidemic. Arch Intern Med. 2003;163:1009–21. 12742798 10.1001/archinte.163.9.1009

[20] Houben RM , Sumner T , Grant AD , White RG . Ability of preventive therapy to cure latent *Mycobacterium tuberculosis* infection in HIV‐infected individuals in high‐burden settings. Proc Natl Acad Sci USA. 2014;111:5325–30. 24706842 10.1073/pnas.1317660111 PMC3986199

[21] Golub JE , Cohn S , Saraceni V , Cavalcante SC , Pacheco AG , Moulton LH , et al. Long‐term protection from isoniazid preventive therapy for tuberculosis in HIV‐infected patients in a medium‐burden tuberculosis setting: the TB/HIV in Rio (THRio) study. Clin Infect Dis. 2014;60:639–45. 25365974 10.1093/cid/ciu849 PMC4366579

[22] Martinson NA , Barnes GL , Moulton LH , Msandiwa R , Hausler H , Ram M , et al. New regimens to prevent tuberculosis in adults with HIV infection. N Engl J Med. 2011;365:11–20. 21732833 10.1056/NEJMoa1005136 PMC3407678

[23] Rowe KA , Makhubele B , Hargreaves JR , Porter JD , Hausler HP , Pronyk PM . Adherence to TB preventive therapy for HIV‐positive patients in rural South Africa: implications for antiretroviral delivery in resource‐poor settings? Int J Tuberc Lung Dis. 2005;9:263–9. 15786888

[24] Salomon JA , Vos T , Hogan DR , Gagnon M , Naghavi M , Mokdad A , et al. Common values in assessing health outcomes from disease and injury: disability weights measurement study for the Global Burden of Disease Study 2010. Lancet. 2013;380:2129–43. 10.1016/S0140-6736(12)61680-8 PMC10782811 23245605

[25] Landmark deal secures significant discount on price of medicine to prevent TB [cited 2019 Dec 12]. Available from: https://www.theglobalfund.org/en/news/2019‐10‐31‐landmark‐deal‐secures‐significant‐discount‐on‐price‐of‐medicine‐to‐prevent‐tb/

[26] World Health Organization . Health service delivery costs. Geneva, Switzerland: WHO; 2008.

[27] Vu L , Waliggo S , Zieman B , Jani N , Buzaalirwa L , Okoboi S , et al. Annual cost of antiretroviral therapy among three service delivery models in Uganda. J Int AIDS Soc. 2016;19:20840. 27443270 10.7448/IAS.19.5.20840 PMC4956730

[28] Vassall A , van Kampen S , Sohn H , Michael JS , John KR , den Boon S , et al. Rapid diagnosis of tuberculosis with the Xpert MTB/RIF assay in high burden countries: a cost‐effectiveness analysis. PLoS Medicine. 2011;8:e1001120. 22087078 10.1371/journal.pmed.1001120 PMC3210757

[29] Bureau of Labor Statistics . CPI inflation calculator [cited 2019 Dec 9]. Available from: https://www.bls.gov

[30] World Health Organization . Macroeconomics and health: an update: increasing investments in health outcomes for the poor: second consultation on macroeconomics and health. Geneva: World Health Organization; 2003.

[31] Patenaude BN , Semali I , Killewo J , Bärnighausen T . The value of a statistical life‐year in Sub‐Saharan Africa: evidence from a large population‐based survey in Tanzania. Value Health Regional Issues. 2019;1(19):151–6. 10.1016/j.vhri.2019.07.009 31494486

[32] Marseille E , Larson B , Kazi DS , Kahn JG , Rosen S . Thresholds for the cost–effectiveness of interventions: alternative approaches. Bull World Health Organ. 2014;15(93):118–24. 10.2471/BLT.14.138206 PMC4339959 25883405

[33] Phillips AN , Cambiano V , Nakagawa F , Revill P , Jordan MR , Hallett TB , et al. Cost‐effectiveness of public‐health policy options in the presence of pretreatment NNRTI drug resistance in sub‐Saharan Africa: a modelling study. Lancet HIV. 2018;5(3):e146–54. 10.1016/S2352-3018(17)30190-X PMC5843989 29174084

[34] Gomez GB , Dowdy DW , Bastos ML , Zwerling A , Sweeney S , Foster N , et al. Cost and cost‐effectiveness of tuberculosis treatment shortening: a model‐based analysis. BMC Infect Dis. 2016;16(1):726. 27905897 10.1186/s12879-016-2064-3 PMC5131398

