# Supplementary material for: Cost‐effectiveness of one month of daily isoniazid and rifapentine versus three months of weekly isoniazid and rifapentine for prevention of tuberculosis among people receiving antiretroviral therapy in Uganda
Source: J Int AIDS Soc. 2020 Oct 18;23(10):e25623. doi: 10.1002/jia2.25623 (PMC7569168; doi:10.1002/jia2.25623)
Supplement: Supplementary file 1 — Appendix S1. The purpose of this appendix is to provide supplementary information on the model structure and the analysis performed. [file JIA2-23-e25623-s001.docx]

**Appendix.**

The purpose of this appendix is to provide supplementary information on the model structure and the analysis performed.

**Model Structure**

The model structure is adapted from a previously published model of cost-effectiveness comparing the outcomes of three months of rifapentine and isoniazid (3HP) versus six to nine months of isoniazid preventive therapy (IPT) among people living with HIV (PLWH) in Uganda [1]. Like the original model, the present model simulates 1000 individuals being treated for HIV and initiating preventive therapy over a time horizon of 20 years. We select a 20-year time horizon as a conservative assumption, so as not to overvalue the prevention of TB in a context where innovations in both TB and HIV treatment may substantially change both life expectancy and current patterns of care. This simplified model is structured as a decision tree paired with a Markov state-transition model. Outcomes are presented in DALYs averted due per 2019 USD. For purposes of simplicity, we assume that the modelled population has, on average, a life expectancy that exceeds the 20-year analytical horizon – such that all individuals in the population contribute 20 years to the analysis. Analyses were performed using Microsoft Excel 2016 (Microsoft, Redmond, Washington) and R version 1.2.1335 (R Foundation for Statistical Computing, Vienna Austria).

On entry into the model, we assume that all 1000 individuals (patients receiving ART) are eligible for TB preventive therapy, regardless of confirmed latent TB infection (LTBI) status. Preventive therapy for PLWH without first testing for LTBI is consistent with standard practice in high-TB burden settings [2], as testing for LTBI is both expensive and may result in missed opportunities to provide TB preventive therapy. Of the original patient cohort, a specified proportion (0.26, based on the estimated prevalence of LTBI in Uganda) are assumed to have LTBI, while the others are LTBI-negative and thus do not receive any benefit from therapy. In both groups, a specified proportion of the population is assumed to experience adverse events of a severity sufficient to warrant discontinuation of preventive therapy. Of those who do not experience such an adverse event (0.95), a proportion either successfully complete the preventive therapy regimen (0.94 for 1HP, 0.74 for 3HP within the reference scenario) or prematurely stop therapy.

We conceptualize “efficacy” as the total potential reduction in TB reactivation that could be achieved, if all prescribed doses were taken by all members of the population (or at least those members with LTBI, as people without LTBI are assumed to receive no benefit). We conceptualize “completion” as the fraction of that total potential reduction that is actually achieved in practice, as a result of taking fewer than the prescribed number of doses. For simplicity, we assume that the relationship between (number of doses taken) and (proportion of efficacy attained) is a linear one. To the extent that this relationship is non-linear (for example, if the first few doses taken provide more-than-proportional benefit, or if the first few doses have less-than-proportional benefit unless consolidated with subsequent doses), our estimates of cost-effectiveness will be somewhat – but not substantially – biased. (See below for more details.)

The decision tree ultimately results in four patient populations:

|  | **LTBI Status** | **Preventive Therapy Status** |
| --- | --- | --- |
| **1** | LTBI Positive | Completed Therapy |
| **2** | LTBI Positive | Did not Complete Therapy |
| **3** | LTBI Negative | Completed Therapy |
| **4** | LTBI Negative | Did not Complete Therapy |

Each of these resulting patient populations subsequently enters the Markov state-transition model, in which individuals transition between four health states: On ART, LTBI; On ART, Active TB; Off ART, LTBI; Off ART, Active TB. Patients within the model move through the health states in time steps of one year over the model’s time horizon of 20 years, at which time the analysis ends. All years of life lost (YLL) and years of life with disability (YLD) are then summed over this 20-year analytic time frame and compared between the scenario assuming 3HP and the scenario assuming 1HP.

**Parameter Assumptions**

*Epidemiological & Health System Values*

Based on constructed trends in the annual risk of infection (estimated using surveys of TB infection in infants) and indirect estimates of annual risk of infection calculated from estimates of smear positive TB prevalence from 1990-2014 (data from the World Health Organization), we assume that the LTBI prevalence in Uganda is 0.26 [3].

The model rate of disengagement from HIV care per year (0.108) is based on the all-cause attrition rates estimated by a systematic review of “retention rates from studies describing observational cohorts from sub-Saharan Africa reporting on adult HIV 1-infected patients initiating first line three-drug ART.” [4] Furthermore, we assume those who disengage from ART do so within the first three years, and as a result carry an increased risk of developing and dying from active TB following disengagement [5,6].

Given the current lack of evidence on completion of 3HP in field settings in sub-Saharan Africa, the model 3HP completion rate (0.74) is based on data from clinical trials on completion of 3HP delivered by self-administered therapy in high-burden settings [7]. Although directly observed therapy results in higher completion rates in some settings, direct observation also incurs substantial additional costs and is unlikely to be logistically feasible (for preventive therapy) in the majority of settings to which this analysis might generalize.

Efficacy of 3HP corresponds to the proportion of TB reactivation prevented in the context of full completion of treatment, and is based on a systematic review and meta-analysis of randomized controlled trials of TB preventive therapy in patients with LTBI. Therefore we assume 3HP efficacy to be 0.90 [7].

*Mortality (Annual Risk)*

We assume within the model that all deaths from active TB occur within the 1-year time step during which active TB develops. This model differentiates between causes of death due to active TB and ART-avertable mortality, incorporating different mortality rates for each of the states in the Markov model: LTBI (on ART), LTBI (off ART), Active TB (on ART), and Active TB (Off ART). These mortality rates are estimated using the mortality rates described below.

Annual mortality for LTBI patients on ART (0.0345) was estimated from a prospective cohort study of over 22,000 Ugandan adults of median age 37 years initiating ART [8]. Annual mortality for LTBI Positive patients off ART (0.1326), was based on a cohort of 19,983 Ugandan adults, ages ranging from 15-59 years [9].

Annual mortality for patients with active TB who are on ART and receiving treatment for TB (0.10) is estimated based on two sources. The first is a study of 1044 Ugandan adults enrolled in home-based ART and screened for TB at baseline. Patients were followed up to determine cause of mortality, for which the estimated effectiveness of ART in reducing TB-related mortality was estimated [10]. This rate was then combined with TB mortality rate data presented by the WHO Global TB Report [11].

Annual mortality rate for patients with active TB, off ART, not receiving treatment for TB (0.81) was estimated based on an analysis of TB notification data, included TB cases, cohort treatment outcomes, and more. These data were collected from peer reviewed literature and databases from WHO, the Joint United Nations Programme on HIV/AIDS (UNAIDS), the US Census Bureau, and the US Centers for Disease Control and Prevention. Within this analysis, the number of TB deaths is estimated based on the case fatality rate [12].

**Section II.** **Model Input Costs**

As mentioned within the manuscript, all model costs are reporting in 2019 USD, adjusted using US CPI.

The unit price of Rifapentine ($0.21 per 150mg tablet) is based on The Global Fund announcement in October 2019 of the reduction in the price of rifapentine by 70% from its then current unit price ($1.00 per 150mg) [13]. We assume the unit price of Isoniazid to be $0.02 per 300mg tablet based on industry price estimates, such as that published by The Global Fund [14].

The cost of an outpatient visit in Uganda ($1.41) was estimated as part of the WHO-CHOICE initiative estimates unit cost values for service delivery at a country and regional level using regression modelling [15]. These values are generated as average service costs, based on country- assumptions around the national health services and capacity. The cost of an outpatient visit includes the cost of personnel, capital, and food, but does not include the cost of drugs or diagnostic tests.

The yearly cost of ART treatment per patient ($192) is estimated based on data collected retrospectively as part of a larger study conducted by three organizations in Uganda. We chose to use the cost results for a rural clinic that is part of the Uganda Cares organization because we felt this best reflected the context of this analysis. This original cost calculation was done using a top down approach. The average cost per ART visit at this rural clinic for 2012 is approximately $24 per visit, which is comprised of personnel costs (8.8%), antiretroviral drugs (65.9%), other drugs, laboratory costs (13%), and administrative costs (7.9%) [16].

The yearly cost of active TB treatment per patient ($218) is based on modeled data from a larger analysis on the cost-effectiveness of an Xpert diagnostic intervention, estimated from a health system perspective using an ingredients approach to estimate the cost per person treated [16]. All patients within this model are assumed to be treated with standard WHO-recommended regimens. Treatment costs include the cost of drugs (estimated using the Global Drug Facility and the MSH International Price Tracker), the unit cost of outpatient visits and the unit cost of hospitalizations[15] . We conservatively assume that individuals who do not complete the prescribed regimen still incur half the costs of the regimen. Our final results are not sensitive to this assumption; for example, if those who do not complete treatment are assumed to incur zero costs, the incremental cost effectiveness ratio of 1HP versus 3HP in the reference scenario changed from $1221 to $1414. Regimen costs can be found in Table S1.

**Section III. Sensitivity Analyses**

In addition to the multi-way sensitivity analysis shown in Figure 2 of the main text, we also performed a series of deterministic one-way sensitivity analyses and a probabilistic sensitivity analysis. We also describe how the assumption of a linear relationship between number of doses completed and regimen efficacy can be evaluated.

*One-Way Sensitivity Analyses*

A one-way sensitivity analysis was conducted to determine the parameters that act as key drivers of outcomes in the model. In this analysis, we varied all model parameters by ±15% of their base value and evaluated the incremental cost-effectiveness ratio (ICER), comparing 1HP to 3HP in the reference scenario (equivalent efficacy of the two regimens, 0.74 completion with 3HP, and 0.94 completion with 1HP [0.20 absolute increase in completion with 1HP]). Under this scenario, as shown in Table 2, the primary ICER estimate was $1221. Figure S2 shows the results of these one-way analyses for the four parameters that were found to be most influential. These results served as the motivation for choosing these four parameters for the multi-way sensitivity analysis shown in Figure 2 of the main text.

*Probabilistic Sensitivity Analyses (PSA)*

A PSA was run for each scenario shown in Tables 2 and 3 in the main text. The goal of this analysis was to understand the potential impact of simultaneous uncertainty in multiple parameters on the final results. For each scenario, we generated 1,000 simulations, each of which involved random selection of a value for each parameter in Table 1 across the statistical distributions shown in Table S2.

The uncertainty interval (lower/upper bound) was selected based on observed/reported data when such data were directly available or assumed to be +/- 15% of the reference value when such data were not available. We used triangular distributions for parameters likely to be estimated by decision-makers in their local setting, beta distributions for other parameters bounded between 0 and 1, and gamma distributions for parameters bounded between 0 and infinity. For the parameters with *triangular* distribution, we applied a symmetric distribution with the lower/upper bound shown, taking the base case value as the mode. Parameters of *Beta(α, β)* were estimated using a method of moments: $\hat{\alpha}=\bar{x}\left( \frac{\bar{x}\left( 1-\bar{x} \right)}{\bar{\nu}}-1 \right),$ $\hat{\beta}=\left( 1-\bar{x} \right)\left( \frac{\bar{x}\left( 1-\bar{x} \right)}{\bar{\nu}}-1 \right)$, if $\bar{\nu}<\bar{x}\left( 1-\bar{x} \right)$, where $\bar{x}$ is the sample mean, and $\bar{\nu}$ is the sample variance. Parameters of *Gamma(k, θ)* were estimated with a method of moments: $Shape \hat{k}= \frac{\bar{x^{2}}}{\bar{\nu}} , Scale \hat{\theta}= \frac{\hat{v}}{\bar{x}}$ where $\bar{x}$ is the sample mean, $\bar{v}$ is the sample variance. For beta and gamma distributions, we calculated the distribution variance by assuming that the upper and lower bounds corresponded to +/- one standard deviation. Parameter values used to define each scenario (LTBI Prevalence, 1HP completion, 1HP efficacy, and price of rifapentine) were held constant in that scenario. The uncertainty ranges shown in Table 2 correspond to the 2.5^th^ and 97.5^th^ percentiles of these probabilistic sensitivity analyses; Figure S3 shows the collection of all simulations on a cost-effectiveness plane where the incremental cost is given on the y-axis and the incremental effectiveness on the x-axis.

*Evaluating the assumption of linear relationship between number of doses completed and regimen efficacy*

In our primary analysis, as described above, we used the assumption of a linear relationship between (doses completed) and (cases of TB reactivation averted) to enable a division of the modeled population into “completers” and “non-completers”. There are no empirical data to explicitly support this assumption, but the impact of this assumption can be assessed by evaluation of Figure 2 in the main text. For example, take the primary scenario in Table 2, corresponding to 1HP efficacy of 0.9 and completion of 0.94 and an incremental cost-effectiveness ratio of $1221. If one were to make the following assumptions instead: (a) a given “non-completion” level includes 33% people who completed zero doses and 67% people who completed 50% of prescribed doses; and (b) completing even 50% of the full prescribed schedule provides full efficacy, then:

“0.94 completion” for 1HP corresponds to 3% of people taking zero doses, 6% taking half of prescribed doses, and 91% taking all doses.

“0.74 completion” for 3HP corresponds to 13% of people taking zero doses, 26% of people taking half of prescribed doses, and 61% of people taking all doses.

The “effective” completion for 3HP would then be 0.87, and the “effective” completion for 1HP would then be 0.97 – with an incremental completion of 0.1. This incremental completion would then correspond to a 1HP completion of 0.84 (0.1 higher than the base value of 0.74 for 3HP) in Figure 2A – an ICER of $2144.

This example illustrates that, absent an assumption about a linear relationship between doses completed and regimen efficacy, one must postulate an alternative relationship, as well as the distribution of patients achieving different levels of completion (and the corresponding efficacy of each completion level). However, it also illustrates that the estimated cost-effectiveness ratio is relatively insensitive to this assumption – even under very strong assumptions in the example above (that 2/3 of all “non-completers” actually completed a sufficient course to provide full efficacy), the ICER varied less than under variation of efficacy, completion, and regimen price considered in Figure 2B.

**Appendix References:**

1. Johnson, Karl T., et al. “Cost-Effectiveness of Preventive Therapy for Tuberculosis With Isoniazid and Rifapentine Versus Isoniazid Alone in High-Burden Settings.” Clinical Infectious Diseases, vol. 67, no. 7, Sept. 2018, pp. 1072–78. DOI.org (Crossref), doi:10.1093/cid/ciy230.
2. “treating without testing is standard practice in high LTBI environments”
3. Houben RM, Dodd PJ. The global burden of latent tuberculosis infection: a re-estimation using mathematical modelling. PLoS Med **2016**; 13:e1002152.
4. Fox MP, Rosen S. Patient retention in antiretroviral therapy programs up to three years on treatment in sub-Saharan Africa, 2007–2009: systematic review. TropMed Int Health **2010** (15 Suppl 1):1–15.
5. Sewankambo NK, Gray RH, Ahmad S, et al. Mortality associated with HIV infection in rural Rakai District, Uganda. AIDS **2000**; 14:2391–400.
6. Suthar AB, Lawn SD, Del Amo J, et al. Antiretroviral therapy for prevention of tuberculosis in adults with HIV: a systematic review and meta-analysis. PLoS Med **2012**; 9:e1001270.
7. Sterling TR, Villarino ME, Borisov AS, et al. Three months of rifapentine and isoniazid for latent tuberculosis infection. N Engl J Med 2011; 365: 2155–66.
8. Mills EJ, Bakanda C, Birungi J, et al. Life expectancy of persons receiving combination antiretroviral therapy in low-income countries: a cohort analysis from Uganda. Ann Intern Med **2011**; 155:209–16.
9. Sewankambo NK, Gray RH, Ahmad S, et al. Mortality associated with HIV infection in rural Rakai District, Uganda. AIDS **2000**; 14:2391–400.
10. Moore D, Liechty C, Ekwaru P, et al. Prevalence, incidence and mortality associated with tuberculosis in HIV-infected patients initiating antiretroviral therapy in rural Uganda. AIDS **2007**; 21:713–9.
11. World Health Organization. 2016 global tuberculosis report. Geneva, Switzerland:WHO, **2016**.
12. Corbett EL, Watt CJ, Walker N, et al. The growing burden of tuberculosis: globaltrends and interactions with the HIV epidemic. Arch Intern Med **2003**; 163:1009–21.
13. Landmark Deal Secures Significant Discount on Price of Medicine to Prevent TB. https://www.theglobalfund.org/en/news/2019-10-31-landmark-deal-secures-significant-discount-on-price-of-medicine-to-prevent-tb/. Accessed 12 Dec. 2019.
14. The Global Fund, Pooled Procurement Mechanism Reference Pricing: Strategic Medicines Used in HIV Programs, 2020. <https://www.theglobalfund.org/media/7500/ppm_strategicmedicineshivreferencepricing_table_en.pdf>.
15. World Health Organization. Health service delivery costs. Geneva, Switzerland: WHO, **2008**.
16. Vu L, Waliggo S, Zieman B, et al. Annual cost of antiretroviral therapy among three service delivery models in Uganda. J Int AIDS Society 2016; 19(5 Suppl4):20840.
17. Vassall A, van Kampen S, Sohn H, et al. Rapid diagnosis of tuberculosis with the Xpert MTB/RIF assay in high burden countries: a cost-effectiveness analysis. PLoS Med 2011; 8:e1001120.
