# Supplementary material for: Cost‐effectiveness of one month of daily isoniazid and rifapentine versus three months of weekly isoniazid and rifapentine for prevention of tuberculosis among people receiving antiretroviral therapy in Uganda
Source: J Int AIDS Soc. 2020 Oct 18;23(10):e25623. doi: 10.1002/jia2.25623 (PMC7569168; doi:10.1002/jia2.25623)

**Table S1. Complete vs. Incomplete Preventive Regimen Costs**

The following table reflects the costs of complete and incomplete preventive therapy regimens, calculated using the cost components described above.

|  |  | **Total Cost of Drug Regimen** | **Total Cost of Drug Regimen Cost with Outpatient Visits** |
| --- | --- | --- | --- |
| **1HP** | Complete | $26.43 | $31.95 |
|  | Incomplete | $13.22 | $15.98 |
| **3HP** | Complete | $17.35 | $28.39 |
|  | Incomplete | $5.78 | $8.54 |

**Figure S1. One-Way Sensitivity Analysis, Impact on ICER (2019 USD per DALYs Averted)**

The following diagram shows the impact of the four most influential drivers of the incremental cost-effectiveness of 1HP (relative to 3HP): 1HP efficacy, 1HP completion rate, the price of rifapentine, and the prevalence of LTBI. Low/high inputs correspond to ±15% of the base parameter value. High value input for 1HP efficacy and 1HP completion of over 1.0 were capped at 1.0. The vertical line corresponds to the primary estimate (seen in Table 2) of $1221 per DALY averted.


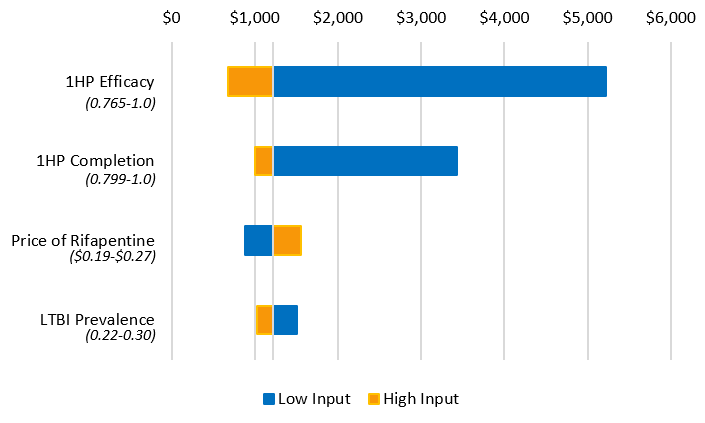


Table S2. Parameter Distributions for Probabilistic Sensitivity Analysis

| **Parameter Description** | **Reference (Model) Value** | **Distribution Type** | **Distribution Parameters** | |
| --- | --- | --- | --- | --- |
| **Epidemiologic and Health System Values** |  |  | Alpha ($\alpha)$ | Beta ($\beta)$ |
| Prevalence of LTBI [15] | 0.261 | Beta | 2.23 | 6.33 |
| Rate of Disengagement from HIV Care, per year [21] | 0.108 | Beta | 7.70 | 63.57 |
|  |  |  | Lower bound | Upper bound |
| Proportion Completing 3HP [9] | 0.74 | Triangular | 0.47 | 0.89 |
| Efficacy of 3HP [9] | 0.9 | Triangular | 0.77 | 1 |
| **Mortality (Annual Risk)** |  |  | Alpha ($\alpha)$ | Beta ($\beta)$ |
| HIV-Positive, on ART [22] | 0.0354 | Beta | 10.31 | 280.91 |
| HIV-Positive, off ART [23] | 0.1326 | Beta | 123.61 | 808.57 |
| Active TB, on ART, Receiving Treatment for TB [24,25] | 0.1 | Beta | 2.03 | 18.25 |
| Active TB, Off ART, Not Receiving Treatment for TB [26] | 0.81 | Beta | 5.01 | 1.17 |
| **Morbidity** |  |  | Alpha ($\alpha)$ | Beta ($\beta)$ |
| Annual Risk of TB Reactivation for PLWH, no ART or TB Preventive Therapy [27] | 0.043 | Beta | 49.11 | 1,092.97 |
| Relative Risk of TB Reactivation while on ART [28] | 0.35 | Beta | 12.03 | 22.34 |
| Prevalence of a Nonlethal adverse Event During TB Preventive Therapy [15,29,30] | 0.034 | Beta | 4.61 | 131.05 |
| **Disability Weights** |  |  | Lower bound | Upper bound |
| Off ART, LTBI [31] | 0.582 | Triangular | 0.406 | 0.743 |
| On ART, LTBI [31] | 0.078 | Triangular | 0.052 | 0.111 |
| Active TB [31] | 0.408 | Triangular | 0.274 | 0.549 |
| **Costs (2019 US Dollars)** |  |  | Shape (k) | Scale ($\theta$) |
| Price of Rifapentine (per 150mg) [13] | $0.21 | Gamma | 43.56 | 0.01 |
| Cost of Outpatient Visit [32] | $1.41 | Gamma | 0.56 | 4.94 |
| Yearly Cost of ART Drugs [33] | $191.81 | Gamma | 79.62 | 2.41 |
| Yearly Cost of Active TB Treatment [34] | $231.02 | Gamma | 21.34 | 10.82 |

**Figure S2. Probabilistic Sensitivity Analyses**

Each panel shows, for one of the scenarios given in Table 2, a series of 1000 dots corresponding to independent simulations in which each model parameter value was selected from a uniform distribution bounded by ±15% of the primary parameter value. The x-axis shows the incremental number of DALYs averted by using 1HP versus 3HP (across a population of 10,000 ART clinic clients offered TB preventive therapy), and the y-axis shows the corresponding incremental cost in 2019 US dollars. The incremental cost-effectiveness ratio for each simulation is therefore given by the slope of the line from the origin to the corresponding point on the graph. The 2.5^th^ and 97.5^th^ percentiles of these simulations are given as uncertainty ranges in Table 2.


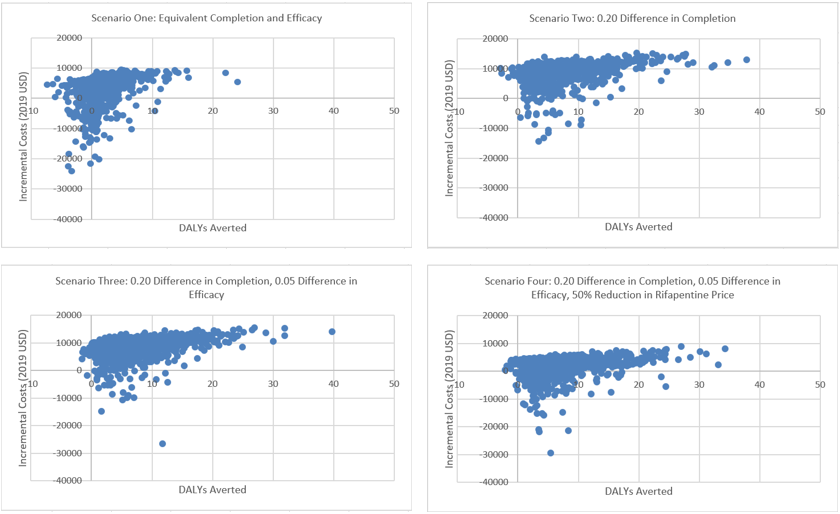

Supplement: Supplementary file 2 — Table S1. Complete versus incomplete preventive regimen costs Table S2. Parameter distributions for probabilistic sensitivity analysis Figure S1. One‐way sensitivity analysis, impact on ICER (2019 USD per DALYs averted). Figure S2. Probabilistic sensitivity analyses. [file JIA2-23-e25623-s002.docx]
